# Supervoxels-Based Histon as a New Alzheimer’s Disease Imaging Biomarker

**DOI:** 10.3390/s18061752

**Published:** 2018-05-29

**Authors:** César A. Ortiz Toro, Consuelo Gonzalo-Martín, Angel García-Pedrero, Ernestina Menasalvas Ruiz

**Affiliations:** Centro de Tecnología Biomédica, Campus de Montegancedo, Universidad Politécnica de Madrid, 28233 Pozuelo de Alarcón, Spain; angel.garcia@ctb.upm.es (A.G.-P.); emenasalvas@fi.upm.es (E.M.R.)

**Keywords:** Alzheimer’s disease, histon, superpixels, supervoxels, MRI, PCA, SVM, classification

## Abstract

Alzheimer’s disease (AD) represents the prevalent type of dementia in the elderly, and is characterized by the presence of neurofibrillary tangles and amyloid plaques that eventually leads to the loss of neurons, resulting in atrophy in specific brain areas. Although the process of degeneration can be visualized through various modalities of medical imaging and has proved to be a valuable biomarker, the accurate diagnosis of Alzheimer’s disease remains a challenge, especially in its early stages. In this paper, we propose a novel classification method for Alzheimer’s disease/cognitive normal discrimination in structural magnetic resonance images (MRI), based on the extension of the concept of histons to volumetric images. The proposed method exploits the relationship between grey matter, white matter and cerebrospinal fluid degeneration by means of a segmentation using supervoxels. The calculated histons are then processed for a reduction in dimensionality using principal components analysis (PCA) and the resulting vector is used to train an support vector machine (SVM) classifier. Experimental results using the OASIS-1 database have proven to be a significant improvement compared to a baseline classification made using the pipeline provided by Clinica software.

## 1. Introduction

Alzheimer’s disease (AD) is the predominant form of dementia in the elderly, and the number of patients is expected to multiply over the next few years [1]. Alzheimer’s disease, at its earliest stage, involves small-scale alterations in the brain defined by the presence of neurofibrillary tangles and beta-amyloid plaque deposits (Aβ) [2]. These alterations result primarily in damage to synapses, followed by degeneration of the axons and, ultimately, atrophy of the dendritic tree and perikaryon and leading to atrophy in specific regions of the brain [3]. This process especially affects specific areas of the brain such as the right and left hippocampus, temporal gyri, cingulate and precuneus [4].

It is becoming increasingly apparent that, when a patient is diagnosed with Alzheimer’s disease, the atrophy is already well established in the brain. The earliest clinical presentation of symptoms that can eventually progress to a clinical diagnosis of Alzheimer’s disease is generally classified as mild amnesic cognitive impairment (MCI) [5]. MCI can be considered a state of clinical impairment, commonly memory loss, beyond that which can be expected for the subject’s age and education without meeting the criteria for classification as dementia. Although not all patients with amnestic MCI will develop AD, cerebral atrophy is already present at this stage [6]. In mildly Alzheimer’s disease affected individuals, entorhinal volumes have already been reduced by 20–30% and hippocampus volumes by 15–25% [7]. Estimates of the progression of atrophy in Alzheimer’s disease cases, between 0.8% and 2% per year [8], suggest that the atrophy process associated with the disease in areas such as the medial temporal lobe must have been active for a period of several years prior to diagnosis or even the presence of symptoms.

The process of degeneration can be visualized through various modalities of medical imaging and has proven to be a valuable biomarker of the stage and potential aggressiveness of the neurodegenerative aspect of Alzheimer’s disease pathology [9]. As a result, in the last two decades, the use of imaging modalities has gone from being a mere secondary instrument in the diagnosis of Alzheimer’s to one of the main tools. Major advances in neuroimaging have provided opportunities to study neurological-related diseases. Thus, resting-state functional magnetic resonance imaging (fMRI) [10], imaging the intrinsic functional brain connectivity, or the use of PET radiotracers developed to allow the in vivo visualization of tau aggregates and Aβ plaques ([11,12]), have become relevant biomarkers for Alzheimer’s disease research. However, despite these advances, FDG positron emission tomography (PET) and magnetic resonance imaging (MRI) are still extensively used in AD-related studies, especially the latter, given its wide availability, non-invasive nature and relative absence of patient discomfort. However, early-stage changes in Alzheimer’s disease are subtle and it is difficult to distinguish patterns by conventional radiological evaluation. Therefore, it remains difficult to establish reliable biomarkers for the diagnosis and monitoring of disease progression, especially in the early stages. This has led to the development of numerous automatic methods for the assessment of brain atrophy.

There is a large body of research published on MRI neuroimaging-based computer-aided classification of AD (see [13] or [14]). Taking into account the work presented in [15], these methods can be grouped into three categories, depending on the type of characteristics that are used to assess the structural variation and how they are extracted. Thus, there are methods based on density maps, either using the whole brain as a unit or relying on a parcellation; methods derived from the study of the cortical surface, also in an overall or local way; and methods based only on the examination of specific regions of the brain.

Density mapping methods look for atrophy patterns by using white matter (WM), grey matter (GM), and cerebrospinal fluid (CSF) mapping generated by voxel-based morphometry (VBM) methods [16]. The direct classification of these features is carried on using either support vector machines (SMV) in [17] and [18] or the programming boosting method (LPBM) [19]. Other works rely on different kinds of feature reduction methods, supervised or unsupervised, in order to reduce the height dimensionality of the feature space. In [20], WM and GM density map dimensions are reduced by the mean of principal component analysis (PCA) and the result is used to train an SVM based classifier. In [21], the reduction in the dimensions of GM maps is proposed only using the intensity distribution of voxels of GM maps as features. Another way of feature selection relies on the use of a cerebral partitioning atlas to obtain regional measurements of the anatomical features to question the presence of abnormal tissue areas as in [22] or [23].

Methods derived from the cortical surface use subtle changes extracted at the vertex-level from a cortical surface, represented primarily as cortical thickness measurements. As in density mapping methods, these measurements can be used directly [24] or processed for the reduction of dimensionality. Some examples on the latter category are found in the work presented in [25] where the cortical surface is modeled using three-dimensional meshes and the cortical thickness is extracted by parametrizing these meshes or the method presented in [26], where thickness data from the cortical surface data are converted into a frequency domain and the dimensionality is reduced by filtering out high-frequency components.

The methods of the third category analyse specific regions in the brain. The main approach in these methods implies the use of biomarkers extracted from the hippocampus [27,28] as volume and/or shape or textural features. Work on other cerebral areas has been done in [29], in which a diffeomorphometry study has been carried out in a number of regions, including the right and left hippocampus, thalamus, and lateral ventricles, in order to perform a linear discriminant analysis for AD prediction.

Related to the methods derived from density maps, but based on the use of textural measurements instead of direct measurements, in this work, we propose the extension of the concept of histons [30] to volumetric images and its use as a textural feature in the classification of T1-weighted MRI images, in order to differentiate Alzheimer’s disease (AD) patients from cognitive normal (CN) patients. Textural methods can identify voxel-intensity patterns and relationships hidden from the unaided human eye [31]. The histon concept represents a way to visualize information about color regions in an image. Compared to other textural characteristics, a histon is particularly sensitive to subtle variations in color in relation to the space [32]. The proposed work offers a simple whole-brain descriptor based on the relationship between the voxel probabilities corresponding to gray matter, white matter or cerebrospinal fluid—instead on a unique voxel intensity measurement as in [33] or [34]. The partitioning of the MRI image into these three probability volumes allows each of them to be equated to a spectral band and to characterize the MRI volume within the RGB color space.

The rest of the paper is organized as follows: Section 2 briefly reviews the concepts underlining this work: histons and supervoxels. The materials used and the general pipeline of the presented method are detailed in Section 3. The experimental results are presented and discussed, in Section 4, concluding in Section 5 with the final remarks and possible future work related to the proposed classification method.

## 2. Background

This section presents the underlying concepts behind our proposal, those related to the superpixel-based segmentation carried out, as well as specific features proposed for Alzheimer’s disease classification.

### 2.1. Superpixel Segmentation and SLIC

A superpixel can be defined as a perceptually uniform region in the image (see [35]). The concept behind the idea of superpixels is the fact that the construction of an image using pixels is simply a technological limitation associated with the image capturing device, not a real property of the source of that image. A superpixel segmentation produces a set of small spectrally-constrained areas in the image, an over-segmentation, which can be used for the estimation of local scale image features. Superpixels capture redundancy in the image and significantly reduce the complexity of the subsequent image processing operations. Since their introduction, superpixels have been successfully used in applications such as pre-processing steps in image segmentation [36,37], depth estimation [38], tracking [39] or skeletonization [40]. Multiple techniques have been developed to generate superpixels. In this work, we will use SLIC. Simple Linear Iterative Clustering (SLIC, [41]) is a spatially constrained revision of the k-means algorithm. It begins by sampling a number of regularly-spaced positions as centres of the clusters, followed by a k-means clustering procedure. SLIC redefines the k-means distance $D_{w}$ by adding a second, colour-based component so the clustering distance between two different pixels is weighted by the colour distance and space distance, defined as:

(1) $$D_{w}=\sqrt{{\left(\frac{d_{c}}{m}\right)}^{2}+{\left(\frac{d_{s}}{In}\right)}^{2}},$$

(2) $$d_{c}=\sqrt{\sum_{s_{i}\in S}{\left(I\left(x_{1},y_{1},s_{i}\right)-I\left(x_{2},y_{2},s_{i}\right)\right)}^{2}}\;\;\;\;\;\;\;\;d_{s}=\sqrt{{\left(x_{1}-x_{2}\right)}^{2}+{\left(y_{1}-y_{2}\right)}^{2}},$$

where both the colour and spatial distance between the pixels $I\left(x_{1},y_{1},s_{i}\right)$ and $I\left(x_{2},y_{2},s_{i}\right)$ in the spectral band $s_{i}$ are represented by $d_{c}$ and $d_{s},$ respectively. Related to the spatial distance, the parameter In is the sampling interval of the cluster’s centroids. Associated with the color distance, the parameter *m* controls the compactness of the superpixels, as the greater the value of *m*, the more the spatial proximity is emphasized. The spatial distance restrictions ensure superpixel homogeneity.

SLIC produces an even superpixel distribution on the image that adheres to the object’s limits as well as or better than the supepixels generated by others methods (see [42]). The SLIC generation algorithm can be easily modified to work on different spatial or spectral domains. Specifically, the extension of these ideas to a volumetric image is called “supervoxel”.

### 2.2. Histon

A histon [43] is a contour added on the top of any of the existing histograms of the spectral components of the image. It exploits co-occurrences between neighbouring pixels, both on the same spectral plane as in adjacent planes, as a method of asserting an intra and extra planar correlation between the components of the image. In this context, a histon can be considered as a textural feature. A histon is defined by a similar colour sphere, known as similarity threshold or expanse, *E*, and a spatial distance measurement $d_{t}\left(x,y\right)$ that defines which pixels should be inserted into each bin of a histogram.

A similarity threshold defines an area in the spectral intensity space in which all the intensity values within this area can be considered part of the same colour value. For an intensity value *g* in the base histogram, this similarity threshold defines the set of points to be evaluated for its pertinence to the corresponding bin in the histogram. Given an image, I(x,y,s) of size M×N, where *s* are the spectral planes in the image, a histon can be expressed as:

(3) $$H_{s_{i}}=\sum_{x=1}^{N}\sum_{y=1}^{M}\left(1+S\left(x,y\right)\right)\delta \left(I\left(x,y,s_{i}\right)-g\right)\;\;\;\mathrm{for}\;0\leq g\leq L-1\;\;\mathrm{and}\;\;s_{i}\in S_{p},$$

where, for each of the spectral components, *L* is the number of intensity levels, $\delta \left(\cdot \right)$ is the Kronecker delta (consequently, $\delta \left(I\left(x,y,s_{i}\right)-g\right)$ that can be seen as a definition of a histogram) and $S\left(x,y\right)$ is a similarity function that tests whether or not an element of the neighbourhood is part of the similar colour sphere (4). If the sum of the spectral distances of the planes is defined as $s_{1},\cdots s_{i},\in S_{p}$ for the neighbourhood of sizes P×Q of any element in the image $\left(x,y\right)$, the distance measurement $d_{t}\left(x,y\right)$ provides the following similarity function:

(4) $$S\left(x,y\right)=\left\{\begin{array}{cc} 1, & \mathrm{if}\;d_{t}\left(x,y\right)<E,\\ 0, & \mathrm{otherwise},\; \end{array}\right.\;\;\;d_{t}\left(x,y\right)=\sum_{p\in P}\sum_{q\in Q}\sqrt{\sum_{s_{i}\in S_{p}}{\left(I\left(x,y,s_{i}\right)-I\left(p,q,s_{i}\right)\right)}^{2}}.$$

In Section 3.3, we propose an extension of the concept of a histon based on a supervoxel segmentation.

The correlation between histogram and histon [43] has been used as an image segmentation method, both for photography [44] and in some modalities of medical imaging [45], including MRI [46]. However, the potential of a histon, in a way a context-aware histogram, as an element of characterization remains essentially unexplored.

## 3. Materials and Methods

**Figure 1:**
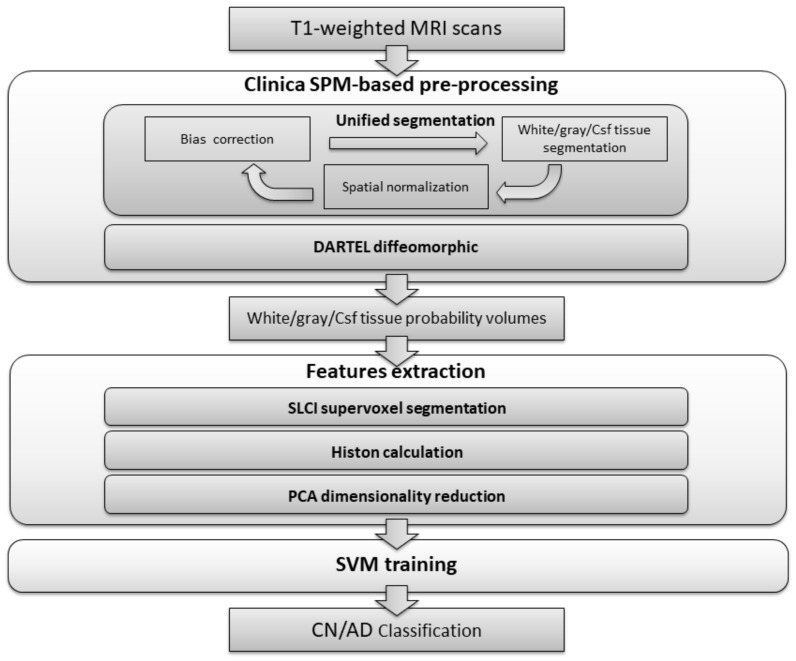
Flowchart of the proposed method.

The classification process presented is divided into three stages, as can be seen in Figure 1. In the first stage, the dataset is processed in order to carry out bias correction and spatial normalization, and to obtain a white matter/grey matter/cerebrospinal fluid segmentation. In the second stage, we calculate a set of histons to be used as a feature vector. To provide a natural neighbourhood for the histon-calculation process, we carry out an over-segmentation using the aggregate volume for GM, WM and CSF. Finally, feature reduction is achieved by means of PCA and the resulting feature vector is used to train an SVM-based classifier.

The subjects included in this study were obtained from the Open Access Series of Imaging Studies (OASIS-1).

### 3.1. Dataset: OASIS

The Open Access Series of Imaging Studies (OASIS) [47] is a project aimed at making MRI data sets of the brain freely available to the scientific community. OASIS is made available from the Washington University Alzheimer’s Disease Research Center, Dr. Randy Buckner at the Howard Hughes Medical Institute (HHMI) at Harvard University, the Neuroinformatics Research Group (NRG) at Washington University School of Medicine, and the Biomedical Informatics Research Network (BIRN).

For this study, the “Cross-sectional MRI Data in Young, Middle-Aged, Non-demented and Demented Older Adults” (OASIS-1) have been selected. This collection is a cross-sectional dataset of 416 individuals of both genders, all right-handed, aged between 18 and 96. The set includes 100 patients (aged over 60) with a clinical diagnosis of Alzheimer’s disease ranging from very mild to moderate. A summary of the demographic characteristics of for the OASIS-1 is shown in Table 1.

### 3.2. Clinica Software

In order to work with a standardized pre-processing work-flow, compatible with multiple neuroimaging databases, the volume pre-processing and general dataset management would be carried out using the Clinica software (version 0.1.0). Clinica is a software platform for clinical neuroscience research studies, developed by the ARAMIS Lab at the Institut du Cerveau et de la Moelle épinière (ICM, Brain & Spine Institute) in Paris, using multimodal data (neuroimaging, clinical and cognitive evaluations, genetics, etc.) and, most often, a longitudinal follow-up.

The general image pre-processing process of a T1-weighted MRI image implies tissue segmentation, bias correction and spatial normalization to the Montreal Neurological Institute (MNI) space. The Clinica software wraps the segmentation procedure from SPM (Statistical Parametric Mapping) [48] that carries out all these processes simultaneously, in a procedure known as “Unified segmentation” [49].

**Figure 2:**
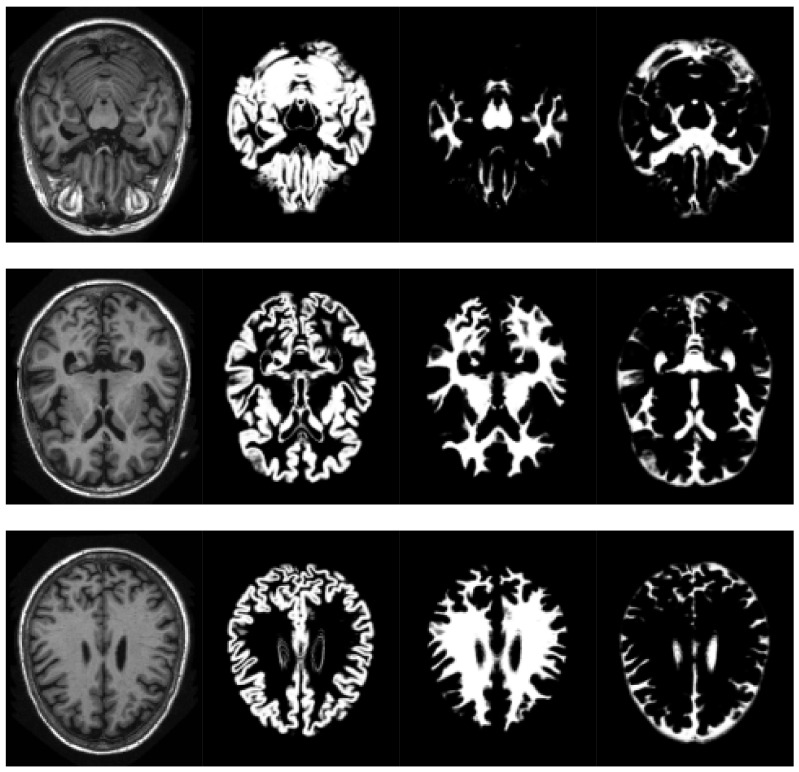
Volume slices, selected from the pre-processed OASIS-1 dataset, showing tissue segmentation examples. Original T1-weighted MRI slice (**left**), gray matter probabilities (**middle left**),white matter probabilities (**middle right**), cerebrospinal fluid (**right**).

SPM models the brain as a layer of cerebrospinal fluid surrounding the gray and white matter. The prior probability that any voxel contains grey or white matter can be determined using a probabilistic atlas of tissue types. The main idea of this method is to model image intensities as a mixture of k Gaussians, where each Gaussian cluster is modelled by its mean, variance and a known tissue mixing proportion. In the unified model, multiple tissue probability maps are used as a priori information of the tissue classes. The Bayes rule is used to produce the posterior probability of each tissue class. This posterior probability is then combined with the data from the image to determine the final tissue type. Using this approach, two voxels with identical intensities can be identified as different tissues. An example of the results obtained can be seen in Figure 2.

The pipeline then computes a group template by applying the DARTEL (Diffeomorphic Anatomical Registration Through Exponentiated Lie. Algebra [50]) diffeomorphic method to the T1-weighted MRI image of each subject considered. In computational anatomy, a diffeomorphic system is a system designed to assign metric distances on the space of anatomical images, in order to permit the quantization and comparison of morphometric changes in anatomical structures. Diffeomorphic mapping is a broad term that may actually refer to a number of different algorithms, processes, and methods. DARTEL is based on the idea of producing a bidirectional “flow field” as the core for image “deformation” in the process of image registering.

The DARTEL process begins by taking the parameter produced by a GM/WM/CSF segmentation and aligning it as close as possible to a set of tissue probability maps, by means of rigid transformations. In the next step, from the average of all the images, an initial template is created that is then used for the simultaneous registration of tissues between images. This model is used to compute individual deformations to each of the individual images, and finally the inverse of the deformations are applied and averaged, in order to regenerate the template. This process is repeated several times. When comparing data from a number of scans, all cerebral volumes are required to be in the same 3D space. In this process, it is achieved by normalizing the volumes on the space defined by the Montreal Neurological Institute (MNI) template.

Finally, Clinica provides a modular way of making a classification based on machine learning by combining different inputs, algorithms, and validation strategies. These modules rely on scikit-learn for classification purposes [51].

### 3.3. Features Extraction: Supervoxel-Based Histons for Structural MRI Alzheimer Detection

As can be seen in [52], in the process of unified segmentation, there is a relationship between neuronal degeneration and the presence of voxels with a relatively low probability of it being part of a specific type of tissue. Since Alzheimer’s disease tends to manifest itself as atrophy in specific areas of the brain, in these cases, the process of tissue segmentation will tend to show “ambiguous” areas difficult to classify as one or the other type of tissue, in addition to the decrease in the total volume of white matter and grey matter associated with ageing. In this work, we propose the possibility of exploiting both the decrease in the total volume of GM and WM and the relationship between GM, WM and CSF through the use of histons as a textural volumetric characteristic.

In [53], we propose the use of super pixel segmentation (using SLIC) as a way of mitigating some of the limitations of the original histon generation method [44] (histons calculated from a color sphere based on a predefined neighbourhood and colour distance), as a method oriented to the segmentation of multispectral images. In this case, we have extended the original method to MRI volumes.

**Figure 3:**
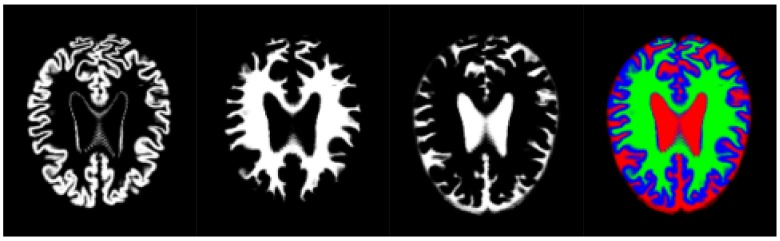
Volume slice from the pre-processed OASIS-1 dataset, showing tissue segmentation probabilities and its corresponding aggregate slice as an RGB image. Gray matter probabilities (**left**), white matter probabilities (**middle left**), cerebrospinal fluid (**middle right**) and aggregate (**right**) (Gray matter probabilities as blue, white matter probabilities as green, cerebrospinal fluid as red).

Thus, we have used an aggregation of the probability volumes for GM, WM and CSF to carry out a segmentation using supervoxels. In this respect, for segmentation purposes, the different probability volumes (which are represented as 8-bit intensity maps) can be equated to the spectral bands of a colour volume (see Figure 3). Using a supervoxel segmentation (over-segmentation), we get a way of characterizing the local similarities within the aggregate volume, obtaining a natural set of neighbourhoods for the generation of histons. Similarly to what can be seen in [53], the use of supervoxels as a neighbourhood implies the adherence of the neighbourhoods set to the boundaries and features present in the image, so, when a histon is calculated, there is a direct spatial relationship between the voxel tested for its belonging and the color sphere. Furthermore, we can quantify the overall local homogeneity of a volume using the average intensity standard deviation of the supervoxel-defined space since segmentation using supervoxels already produces locally homogeneous areas.

We will associate intensity with the probability of it being GM, WM and CSF in each of the probability volumes. Thus, when calculating a histon, a voxel will be considered to be inside the color sphere if the distance between the mean intensity of its corresponding supervoxel and the intensity of that voxel is less than the mean local deviation of the probability volume in the space defined by the supervoxels, in each of the probability volumes. Let us denote the set of resulting supervoxels in an image segmentation as $Cs=\{Cs_{1},Cs_{2},\cdots ,Cs_{Ns}\}$, where Ns is the total number of supervoxels; $I(x_{Cs_{j}},y_{Cs_{j}},z_{Cs_{j}},v_{i})$ represents the centroid of the supervoxel $Cs_{j}$ in the probability volume $v_{i}\in V_{p}$, where $V_{p}$ is the set of probability volumes $V_{p}=\left\{WM,GM,CLF\right\}$; and $Np_{j}$ is the number of voxels in that supervoxel. Then, the similarity function $S_{2}\left(x,y,z\right)$ is defined as:

(5) $$S_{2}\left(x,y,z\right)=\left\{\begin{array}{cc} 1, & \mathrm{if}\;\forall s_{i}\in V_{p}\;\;d_{ts_{i}}\left(x,y,z\right)<E_{v_{i}},\\ 0, & \mathrm{otherwise}, \end{array}\right.$$

(6) $$d_{ts_{i}}\left(x,y,z\right)=\left|I\left(x,y,z,v_{i}\right)-I\left(x_{Cs_{j}},y_{Cs_{j}},v_{i}\right)\right|\;\;\;I\left(x,y,z,v_{i}\right)\in Cs_{j},$$

(7) $$E_{v_{i}}=\frac{1}{Ns}\sum_{j=1}^{Ns}\frac{1}{Np_{j}}\sum_{I\in Cs_{j}}I\left(x,y,z,v_{i}\right).$$

The use of supervoxels as a neighbourhood allows the probability distribution to be represented in a natural and accurate way as a supervoxel represents a real volume within the image, created by taking into account the features present in the image. Thus, the histons will encode the relationships between the probabilities of their being GM, WM and CFS by taking into account their spatial distribution in a volume, based on a natural set of neighbourhoods.

The feature vector obtained consists of 768 components (a different histon for each probability volume, each with 256 levels).

Working with high-dimensional feature vectors makes a classifier prone to over-fitting by choosing the wrong dimension as a discriminatory feature. To decrease the high dimensionality represented by the aggregate histon vector, principal component analysis (PCA) is used. PCA [54] aims to transform a set of original variables into a new set of variables, a linear combination of the original ones, called principal components (PCs), without losing any information. For a standardized dataset, the principal components can be calculated as the normalized eigenvectors of the covariance matrix of the original variables and can be sorted by the amount of variation found in the data they explain. From a geometrical point of view, each component can be viewed as the maximizing direction of the variance of the samples, uncorrelated to previous components, when they are projected onto the component itself. The number of components extracted is equal to the number of variables being analysed, so only a subset of them are generally used in the classification. Usually, only the first few components account for meaningful amounts of variance, and the rest will tend to represent only trivial amounts of variance.

### 3.4. SVM Classifier

To carry out the categorization of the T1-weighted MRI volumes, in order to separate Alzheimer’s disease patients and cognitive normal patients, the Support Vector Machine (SVM) [55] has been selected to train the classifier, as it generally yields good results and is remarkably robust to model bias or model variance [56].

SVM is a general supervised learning method able to carry out binary group separation. An SVM belongs to the category of linear classifiers, as the classification is carried out by finding the plane or (depending on the dimensionality of the problem) hyperplane that better differentiates the two classes. The idea is to obtain what is called a maximum margin on each side of the hyperplane by selecting an equidistant separation hyperplane from the closest samples of each class. Only the data that define the borders (the support vectors) of those margins are considered. The search for the separation hyperplane in these spaces, normally of very high dimension, will be implicitly made using the so-called kernel functions. From an algorithmic point of view, the problem of optimizing the geometric margin represents a quadratic optimization problem with linear constraints that can be solved using standard quadratic programming techniques.

## 4. Results

The experiments were carried out in a subset of the T1-weighted MRI transversal image part of the OASIS-1 dataset, using all the subjects aged 60 and over. To assess the differences in demographic and clinical characteristics between groups (AD and CN), we used a Student’s *t*-test for age and MMSE (Mini-Mental State Examination) and Pearson’s chi-square test for gender. The significance level was set at 0.05. Significant differences between controls and patients were found for the MMSE, and gender. The gender differences between groups, as well as the general large variability in age of the dataset, are factors that can result in a bias in the classification results. Taking this into account, a second reduced dataset will be used, their subjects selected randomly under the criteria of minimizing gender differences between groups and, as far as possible, discarding outliers to decrease standard deviation of the age. A summary of the subject’s demographics and dementia status for the population of both subsets is detailed in Table 2. Segmentation and feature extraction is carried out with our own implementation of SLIC on MATLAB (MATLAB and Statistics Toolbox Release 2017b, The MathWorks, Inc., Natick, MA, USA), feature reduction and classification is carried out using R.

### 4.1. Validation Strategy

In order to obtain unbiased estimates of the performances, following the recommendations presented in [57], each dataset is randomly split ten times into two groups: training sets and testing sets (80/20%). The split division process preserves the distribution of age and gender. Each training set is used to train a classifier, and their corresponding testing sets are used for evaluation purposes. The training sets obtained from the aged 60 and over dataset are also used to determine the optimal kernel for the SVM classifier. Individual demographic information for each split can be seen in Appendix A.

As performance measurement, we report the accuracy (Acc=(TP+TN)/(P+N)), negative prediction value (PPV=TN/(TN+FN)), positive prediction value (PPV=TP/(TP+FP)), sensitivity (Sen=TP/(TP+FN)), specificity (Spe=TN/(TN+FP)), F-score (Fsco=2×((Sen∗PPV)/(Sen+PPV))) and balanced accuracy (Bacc=(Spe+Sen/2)), where *P* is the total number of Alzheimer’s disease patients in the dataset, *F* is the cognitive normal patients in the dataset, true positives (TP) are the correctly classifies Alzheimer’s disease patient volumes, true negatives (TN) are the correctly classified number of cognitive normal volumes, false positives (FP) represent uncorrected classified volumes as cognitive normal patients and (FN) are cognitive normals classified as Alzheimer’s disease patients.

For comparison purposes, the results provided by the machine learning-based classification modules are used as a baseline, following the lines proposed in [58]. In this specific case, DARTEL-modulated gray matter probability maps obtained from the T1-weighted MRI images are used to calculate a linear kernel using the Gram matrix from the feature vectors of the subjects provided (all the voxels in the volume). This kernel is used as input for a generic SVM whose cost parameter is optimized to improve the balanced accuracy by means of an exhaustive grid search. This process is repeated on both aged over 60 and reduced datasets, for each split.

### 4.2. SVM Parameters and Kernel Selection

**Figure 4:**
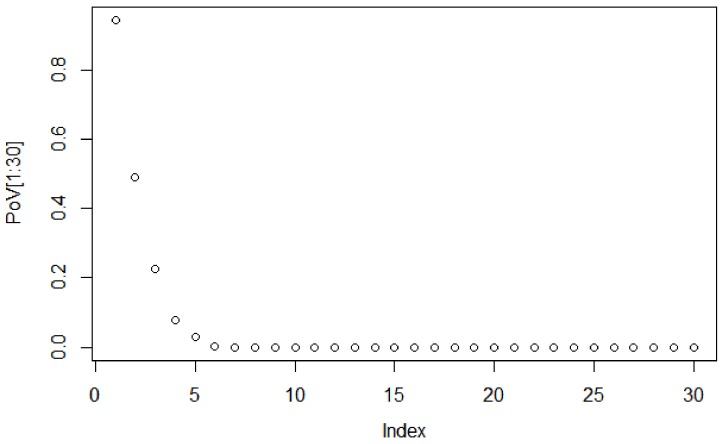
Scree plot of eigenvalues for the first 30 components.

To decide the suitable size of the PCA-based dimensionality reduction, the scree graph method is used. The scree graph [59] shows the the eigenvalues of the covariance against number of principal components. The scree test is used to decide on the size of the feature vector via visual analysis, by looking for a “break” between the components with relatively large eigenvalues and those with small eigenvalues. When the curve bends displaying an “elbow”, it is assumed that the variance explained will not increase significantly with the addition of mere eigenvectors. Figure 4 shows the scree graph, plotting the variance explained in terms of the first 30 main components of the dataset analysed. As can be seen, the elbow occurs between the 5th and 6th principal components; therefore, the first five components appear to be enough to describe the variance in the data.

Since we do not know the specific characteristics of the processed dataset and, therefore, we do not know which is the most appropriate kernel, generic linear, polynomial and radial kernels for the SVM classifier are tested. We apply a 10-fold cross-validation methodology, repeating the folding experiment 10 times for a total of 100 iterations of the algorithm for each of the training subsets of the dataset of subjects aged 60 and over.

Adjusting the parameters in an SVM classifier represents a compromise between achieving the model that best fits the training set and maintaining the classifier ability to generalize to new data (see [60]). A process of parameter optimization (model fitting) can lead to a hyperplane too focused on classifying each element of the training set correctly, resulting in a loss of generalization properties. While this does not have to result in an overfitting problem, it is a possibility that should be taken into account, so no optimization step is carried out on the presented models. The default parameters for the SVM in R are used ( 1 for the cost parameter, 1/(datadimension) for the gamma parameter and 3 for the degree parameter). From the results presented in Table 3, we will select a linear kernel to carry out the rest of the experiments, as it represents an improvement all the performance measurements evaluated.

### 4.3. Classification

Table 4 shows the means of the results obtained with the dataset of subjects aged 60 and over (upper table) as well as with the reduced subset (lower table) using both the Clinica baseline and the proposed histon-based feature classification methods. Individual evaluation results for each split can be located in Appendix B.

To assess whether the proposed method performs significantly better than the Clinica baseline classifier, we used McNemar’s chi-square tests. The use of histons as features represents an improvement in all the proposed evaluation metrics (McNemar test *p* < 0.05 for all splits, except split 5). As can be appreciated from both the confusion matrix (see Table 4, upper row) and the negative and positive prediction values (NPV and PPV), the errors of classification in the Clinica baseline have a slight bias towards false negatives, whereas, for the proposed method, errors are mainly associated with false positives. It should be noted the remarkable differences between the results presented in Table 3 and Table 4 for the aged 60 and over dataset. This is the result of the different evaluation strategies between the two cases. Cross-validation not only has a pessimistic bias (see [57]), but the cross-validation folds are completely random, not respecting the age or gender distribution of the original dataset.

As expected, using the reduced dataset without the bias imposed by significant differences in gender between AD and CN groups and less variability in age, the results improve for both the Clinica baseline and the proposed method, as can be seen in Table 4 (lower table). In this case, the proposed method provides better results for all evaluation metrics compared to the results with the aged 60 and over dataset. The distribution of errors does not differ from the previous scenario (see Table 5, lower row). In this case, we can not claim significance for the results using the McNemar test.It should be noted that the McNemar’s chi-square test may be inadequate for small sample sizes [61].

## 5. Conclusions

In this study, we propose the use of histons as a textural characteristic to carry out the categorization of T1-weighted MRI volumes, in order to separate Alzheimer’s disease and cognitive normal patients. Specifically, Clinica software is used to carry out a preprocessing stage: tissue segmentation, bias correction and spatial normalization to MNI space. After the normalization stage, we perform an over-segmentation using the aggregate volume for gray matter, white matter and cerebrospinal fluid, in order to provide a natural set of neighbourhoods for the histon-calculation process. Then, a subset of the vectors features is selected using PCA. Finally, we train SVM classifiers using the reduced features.

The use of a volume-based histon aims to exploit the relationship between gray matter, white matter and cerebrospinal fluid. For this purpose, the method for histon calculation presented in [53] has been extended to volumetric images. The concept of histons represents a mean for visualization of color information for the evaluation of similar color regions in an image. Compared to other textural features, a histon is especially sensitive to subtle variations of color in relation to space, particularly when we provide a natural set of neighbourhoods for its creation through the use of supervoxels. This allows for quantifying the colour variations associated with neuronal degeneration on a RGB interpretation of an aggregation of the probability volumes for GM, WM and CSF.

Experimental results, on both the aged 60 and over and the reduced subset, have demonstrated a significant improvement in performance for AD versus CN classification compared to the direct voxel classification of the T1-weighted MRI volumes (baseline provided by Clinica). Although given the differences in age, gender, impairment and/or image quality between study populations it is impossible to make a direct comparison, in general, we can affirm that the results obtained are comparable to or better than those of similar textural methods (see [33,62] or [34]). On the other hand, the current implementation of the method, where histons are calculated on the whole brain, does not assert specific spatial patterns of cerebral degeneration associated with Alzheimer’s disease. This may limit the method’s ability to discriminate in the presence of cerebral atrophy associated with other pathologies, or even in very elderly patients.

The use of a standardized work-flow, provided by Clinica, represents an important step towards the reproducibility of this research and its comparability with future developments. However, it should be noted that this pre-processing step can be computationally expensive, especially if large datasets are considered.

The remarkable results obtained with the method proposed suggest the extension of the study to other cases, such as discrimination between cognitive normal and mild cognitive disorder or to predict the evolution from mild cognitive disorder to Alzheimer’s disease, as well as to the expansion and refinement of the study group by extending it to other databases. In the future, it is planned to improve the discrimination capacity of the textural feature presented by applying it only to specific areas of the brain.

## Figures and Tables

**Table 1:** Demographic characteristics of the population studied (OASIS-1 database). Clinical Dementia Rating scale (CDR) values are indicated as the number of patients diagnosed as very mild dementia/mild dementia/moderate dementia. Mini-Mental State Examination (MMSE) values are indicated as mean ± standard deviation.

		Non-Dementia					Dementia				
Age Group	N	n	b	M/F	CDR	MMSE	n	Mean	M/F	CDR	MMSE
<20	19	19	18.53	10/9			0	0	0	0/0/0	
20 s	119	119	22.82	51/68			0	0	0	0/0/0	
30 s	16	16	33.38	11/5			0	0	0	0/0/0	
40 s	31	31	45.58	10/21		29.5 ± 1.5	0	0	0	0/0/0	
50 s	33	33	54.36	11/22		29.4 ± 0.8	0	0	0	0/0/0	
60 s	40	25	64.88	7/18		29.1 ± 1.3	15	66.13	6/9	12/3/0	22.9 ± 5.5
70 s	83	35	73.37	10/25		29.1 ± 1	48	74.42	20/19	32/15/1	24.6 ± 4
80 s	62	30	84.07	8/22		28.7 ± 1.2	32	82.88	13/19	22/9/1	24.5 ± 3.9
90 s	13	8	91.00	1/7		28.3 ± 1.6	5	92.00	2/3	4/1/0	23.8 ± 1.9
	416	316		119/197		29.1 ± 1.1	100		41/59	70/28/2	24.3 ± 4.16

**Table:** 

Diagnosis	Number	Age	Gender	Mean MMSE	CDR
Alzheimer’s disease	100	76.6 ± 7.77	41/59	24.3 ± 4.16	70/28/2
Cognitive normal	98	75.9 ± 8.07	26/72	29.93 ± 3.8	

**Table:** 

Diagnosis	Number	Age	Gender	Mean MMSE	CDR
Alzheimer’s disease	70	75 ± 5.5	23/47	24.8 ± 4.1	49/20/1
Cognitive normal	69	75 ± 4.47	25/44	29.04 ± 3.77	

**Table 3:** Evaluation results showing a comparison between the mean evaluation metrics proposed from the use of different kernels (linear, polynomial and radial) for the SVM classifier for the dataset of aged 60 and over, for the 10 repetitions. The best results are highlighted in bold.

	Accuracy	B. Accu.	NPV	PPV	Sensit.	Specif.	F-Score
Histon: SVM linear kernel	**0.6918**	**0.6913**	**0.7473**	**0.6562**	**0.8012**	**0.5813**	**0.7214**
Histon: SVM polynomial kernel	0.5995	0.5989	0.6181	0.5897	0.6760	0.5217	0.6266
Histon: SVM radial kernel	0.6503	0.65004	0.7034	0.6187	0.7738	0.5262	0.6874

**Table:** 

	Accuracy	B. Accu.	NPV	PPV	Sensit.	Specif.	F-Score
Clinica:Voxel as feature	0.6643	0.6730	0.6415	0.7045	0.615	0.7241	0.6481
Histon: SVM linear kernel	0.8071	0.8001	0,8226	0.7838	0.8541	0.7620	0.8158

**Table:** 

	Accuracy	B. Accu.	NPV	PPV	Sensit.	Specif.	F-Score
Clinica:Voxel as feature	0.7089	0.7234	0.7088	0.7168	0.6845	0.7355	0.6925
Histon: SVM linear kernel	0.8366	0.8334	0.8637	0.8242	0.8783	0.7885	0.8475

**Table:** 

	Predicted Value		
Actual value		AD	CN
	AD	62.7	30.5
	CN	37.2	69.4

**Table:** 

	Predicted Value		
Actual value		AD	CN
	AD	83.7	23.9
	CN	16.2	76.1

**Table:** 

	Predicted Value		
Actual value		AD	CN
	AD	66.2	25.8
	CN	33.3	71.1

**Table:** 

	Predicted Value		
Actual value		AD	CN
	AD	87.9	21.6
	CN	12.1	78.3

## Appendix A. Demographic Information for the Splits Used in the Validation Process

**Table A1:** Demographic information for each split of the aged 60 and over dataset. Gender is indicated as male/female, the Clinical Dementia Rating scale (CDR) values are indicated as the number or patients diagnosed as very mild dementia/mild dementia/moderate dementia. Age and Mini-Mental State Examination (MMSE) values are indicated as mean ± standard deviation.

		Training Set					Testing Set				
	Diag.	N.	Age	Gender	MMSE	CDR	N.	Age	Gender	MMSE	CDR
Split 1	AD	76	76.4 ± 6.89	29/47	24.43 ± 4.45	56/18/2	24	77.7 ± 7.84	12/12	23.3 ± 3.14	14/10/0
	CN	80	75.2 ± 8.62	23/57	29.02 ± 1.17		18	79.11 ± 10.09	3/15	28.66 ± 1.32	
Split 2	AD	74	76.6 ± 6.85	31/43	24.5 ± 3.98	55/18/1	26	77.1 ± 7.96	10/16	23.76 ± 4.67	15/10/1
	CN	82	76.59 ± 8.48	21/61	28.92 ± 1.17		16	72.43 ± 10.87	5/11	29.06 ± 1.38	
Split 3	AD	80	76.42 ± 7.52	33/47	24.4 ± 4.19	58/21/1	20	78.1 ± 5.14	8/12	23.85 ± 4.14	12/7/1
	CN	70	75.46 ± 8.91	20/56	28.97 ± 1.25		22	77.5 ± 9.25	6/16	28.9 ± 1.06	
Split 4	AD	79	76.3 ± 6.59	36/43	24.64 ± 4.07	56/21/2	21	77.7 ± 8.94	5/16	23.09 ± 4.35	14/7/0
	CN	77	75.88 ± 9.36	19/58	23.09 ± 1.11		21	76.047 ± 7.04	7/14	28.55 ± 1.46	
Split 5	AD	78	77.28 ± 7.46	30/48	23.93 ± 4.28	52/24/2	22	74.9 ± 5.5	11/11	25.68 ± 3.46	18/4/0
	CN	78	76.14 ± 8.82	21/57	28.91 ± 1.23		20	75.05 ± 9.76	5/15	29.15 ± 1.8	
Split 6	AD	84	76.73 ± 6.83	33/51	24.57 ± 4.02	62/21/1	16	76.87 ± 8.73	8/8	23 ± 4.77	8/7/1
	CN	72	75.77 ± 9.42	21/51	29.02 ± 1.11		26	74.3 ± 7.8	5/21	28.76 ± 1.45	
Split 7	AD	80	77.11 ± 7.25	35/45	24.52 ± 4.1	57/23/0	20	75.35 ± 6.53	6/14	23.5 ± 4.39	13/5/2
	CN	76	76.22 ± 9.03	19/57	28.93 ± 1.21		22	74.86 ± 8.92	7/15	29.04 ± 1.21	
Split 8	AD	77	76.55 ± 7.31	31/46	23.92 ± 4.39	51/24/2	23	77.43 ± 6.51	10/13	25.65 ± 2.99	19/4/0
	CN	79	75.6 ± 9.28	21/58	29 ± 1.23		19	77.21 ± 7.72	5/14	28.78 ± 1.13	
Split 9	AD	76	76.94 ± 7.52	34/42	24.94 ± 3.77	57/18/1	24	76.1 ± 5.75	7/17	22.33 ± 4.77	13/10/1
	CN	80	75.9 ± 8.72	21/59	28.96 ± 1.26		18	79.94 ± 10.33	5/13	28.94 ± 0.93	
Split 10	AD	81	76.7 ± 7.34	31/50	24.25 ± 4.17	56/25/0	19	77 ± 6.23	10/9	24.57 ± 3.14	14/3/2
	CN	75	76.12 ± 9.17	19/56	29 ± 1.2		23	75.26 ± 8.49	7/16	28.82 ± 1.23	

**Table A2:** Demographic information for each test split of the reduced dataset. Gender is indicated as male/female, the Clinical Dementia Rating scale (CDR) values are indicated as the number or patients diagnosed as very mild dementia/mild dementia/moderate dementia. Age and Mini-Mental State Examination (MMSE) values are indicated as mean ± standard deviation.

		Training Set					Testing Set				
	Diag.	N.	Age	Gender	MMSE	CDR	N.	Age	Gender	MMSE	CDR
Split 1	AD	54	75.88 ± 4.87	20/24	24.37 ± 3.79	38/16/0	16	75.87 ± 4.55	8/8	25.5 ± 5.03	11/4/1
	CN	55	75.1 ± 6.80	22/33	28.9 ± 1.33		14	73.92 ± 5.26	3/11	29.13 ± 0.66	
Split 2	AD	56	75.85 ± 4.73	21/35	24.92 ± 3.83	40/16/0	14	76 ± 5.11	7/7	23.42 ± 5	9/4/1
	CN	53	74.73 ± 6.15	21/32	28.96 ± 1.25		16	75.31 ± 7.76	4/12	28.93 ± 1.18	
Split 3	AD	53	75.77 ± 5.04	21/32	24.11 ± 4.13	35/17/1	17	75.16 ± 6.36	7/10	26.23 ± 3.61	14/3/0
	CN	56	75.16 ± 6.36	22/34	28.89 ± 1.26		13	73.61 ± 7.19	3/10	29.23 ± 1.09	
Split 4	AD	56	75.82 ± 5	22/34	24.5 ± 4.24	38/17/1	14	76.14 ± 3.86	6/8	25.14 ± 3.54	11/3/0
	CN	53	75.5 ± 6.33	20/33	29.05 ± 1.19		16	72.75 ± 6.8	5/11	28.62 ± 1.31	
Split 5	AD	53	75.9 ± 4.68	20/33	24.33 ± 3.68	36/16/1	17	75.58 ± 5.17	8/9	25.52 ± 5.22	13/4/0
	CN	56	75.39 ± 5.17	21/35	28.92 ± 1.26		13	72.61 ± 5.82	4/9	29.07 ± 1.11	
Split 6	AD	52	75.67 ± 5.03	20/32	24.11 ± 4.07	35/16/1	18	76.5 ± 3.98	8/10	26.11 ± 3.9	14/4/0
	CN	57	74.94 ± 6.43	20/37	28.87 ± 1.29		12	74.5 ± 7.11	5/7	29.33 ± 0.77	
Split 7	AD	54	76.07 ± 5	21/33	25.31 ± 4.02	41/12/1	16	75.25 ± 3.99	7/9	22.31 ± 3.57	8/8/0
	CN	55	74.76 ± 6.5	19/36	28.98 ± 1.19		14	75.28 ± 6.74	6/8	28.85 ± 1.4	
Split 8	AD	55	75.87 ± 4.58	24/31	24.96 ± 3.76	40/15/1	15	76.93 ± 5.59	4/11	23.4 ± 5.12	9/5/0
	CN	54	74.64 ± 5.59	19/35	28.88 ± 1.25		15	74.64 ± 6.54	6/9	29.2 ± 1.14	
Split 9	AD	54	76.18 ± 4.98	24/30	24.7 ± 3.97	37/16/1	16	74.87 ± 3.96	4/12	24.37 ± 4.61	12/4/0
	CN	55	74.3 ± 6.14	19/36	28.98 ± 1.2		14	73.14 ± 7.76	6/8	28.65 ± 1.35	
Split 10	AD	60	75.76 ± 4.5	27/33	24.81 ± 4.05	42/18/0	10	76.6 ± 6.34	1/9	23.5 ± 4.4	6/3/1
	CN	49	74.26 ± 6.35	15/34	29.04 ± 1.11		20	76.35 ± 6.8	10/10	28.75 ± 1.48	

## Appendix B. Individual Evaluation Results for Each Data Split

Table A3 Evaluation results showing a comparison between the evaluation metrics for each test split using the Clinica baseline (**upper table**) and the proposed method (SVM trained using a linear kernel) for the dataset of subjects aged 60 and over (**lower table**).

**Table:**

	Accuracy	B. Accu.	NPV	PPV	Sensit.	Specif.	F-Score
Split 1	0.619	0.6082	0.5625	0.6538	0.7083	0.5	0.68
Split 2	0.6667	0.7321	0.5357	0.9286	0.5	0.9375	0.65
Split 3	0.7143	0.7159	0.75	0.6818	0.75	0.6818	0.7143
Split 4	0.6905	0.7019	0.6538	0.75	0.5714	0.8095	0.6486
Split 5	0.7381	0.7426	0.6957	0.7895	0.6818	0.8	0.7317
Split 6	0.5476	0.5366	0.6522	0.4211	0.5	0.5769	0.4571
Split 7	0.6905	0.6905	0.7143	0.6667	0.7	0.6818	0.6829
Split 8	0.6429	0.6528	0.5833	0.7222	0.5652	0.7368	0.6341
Split 9	0.6429	0.6624	0.56	0.7647	0.5417	0.7778	0.6341
Split 10	0.6905	0.6875	0.7083	0.6667	0.6316	0.7391	0.6486

**Table:**

	Accuracy	B. Accu.	NPV	PPV	Sensit.	Specif.	F-Score
Split 1	0.7857	0.7777	0.7647	0.8	0.8333	0.7222	0.8163
Split 2	0.8095	0.81	0.7222	0.875	0.8076	0.8125	0.84
Split 3	0.8333	0.8409	1	0.7407	1	0.6818	0.8510
Split 4	0.7857	0.7857	0.8	0.7727	0.8095	0.7619	0.7906
Split 5	0.7857	0.7886	0.7391	0.8421	0.7272	0.85	0.7804
Split 6	0.8095	0.8341	0.95	0.6818	0.9375	0.7307	0.7894
Split 7	0.8095	0.8113	0.85	0.7727	0.85	0.7727	0.8095
Split 8	0.7857	0.7814	0.7778	0.7916	0.826	0.7368	0.8085
Split 9	0.7857	0.7708	0.8	0.7778	0.875	0.6667	0.8235
Split 10	0.7857	0.7708	0.8	0.7778	0.875	0.6667	0.8235

Table A4 Evaluation results showing a comparison between the evaluation metrics for each test split, using the Clinica baseline (**upper table**) and the proposed method (SVM trained using a linear kernel) for the reduced dataset (**lower table**).

**Table:**

	Accuracy	B. Accu.	NPV	PPV	Sensit.	Specif.	F-Score
Split 1	0.7	0.7098	0.6315	0.8181	0.5625	0.8571	0.6667
Split 2	0.63333	0.6205	0.6190	0.6666	0.4285	0.8125	0.5217
Split 3	0.7	0.8597	0.625	0.7857	0.6470	0.7692	0.7097
Split 4	0.7333	0.74107	0.8333	0.6666	0.8571	0.625	0.75
Split 5	0.8	0.7964	0.8235	0.7692	0.7692	0.8235	0.7692
Split 6	0.7667	0.7579	0.8235	0.6923	0.75	0.7778	0.72
Split 7	0.7	0.6964	0.6923	0.70588	0.75	0.6428	0.7273
Split 8	0.6897	0.6827	0.6154	0.75	0.7059	0.6667	0.7273
Split 9	0.7	0.7	0.8	0.6	0.75	0.6667	0.6667
Split 10	0.6666	0.6696	0.625	0.7142	0.625	0.7142	0.6667

**Table:**

	Accuracy	B. Accu.	NPV	PPV	Sensit.	Specif.	F-Score
Split 1	0.8666	0.8660	0.8571	0.875	0.875	0.8571	0.875
Split 2	0.8333	0.8303	0.8235	0.8461	0.7857	0.875	0.8148
Split 3	0.8333	0.8348	0.7857	0.875	0.8235	0.8461	0.8484
Split 4	0.8666	0.875	1	0.7777	1	0.75	0.875
Split 5	0.8	0.7873	0.8181	0.7894	0.8823	0.6923	0.8333
Split 6	0.8333	0.8333	0.7692	0.8823	0.8333	0.8333	0.8571
Split 7	0.8333	0.8258	0.9090	0.7894	0.9375	0.7142	0.8571
Split 8	0.8667	0.8611	0.8889	0.8333	0.8333	0.8889	0.8333
Split 9	0.8	0.7991	0.7857	0.8125	0.8125	0.7857	0.8125
Split 10	0.8812	0.8718	0.921	0.8238	0.875	0.7727	0.8485
